# Translation, cultural adaptation and validation of the Diabetes Attitudes Scale - third version into Brazilian Portuguese ^1^

**DOI:** 10.1590/1518-8345.1404.2875

**Published:** 2018-01-08

**Authors:** Gisele de Lacerda Chaves Vieira, Adriana Silvino Pagano, Ilka Afonso Reis, Júlia Santos Nunes Rodrigues, Heloísa de Carvalho Torres

**Affiliations:** 2 Doctoral student, Escola de Enfermagem, Universidade Federal de Minas Gerais, Belo Horizonte, MG, Brazil.; 3 PhD, Full Professor, Faculdade de Letras, Universidade Federal de Minas Gerais, Belo Horizonte, MG, Brazil.; 4 PhD, Adjunct Professor, Instituto de Ciências Exatas, Universidade Federal de Minas Gerais, Belo Horizonte, MG, Brazil.; 5 Undergraduate student in Language and Literature, Faculdade de Letras, Universidade Federal de Minas Gerais, Belo Horizonte, MG, Brazil.; 6 PhD, Associate Professor, Escola de Enfermagem, Universidade Federal de Minas Gerais, Belo Horizonte, MG, Brazil.

**Keywords:** Translating, Surveys and Questionnaires, Diabetes Mellitus, Health Knowledge, Attitudes, Practice, Validation Studies, Reproducibility of Results

## Abstract

**Objective:**

to perform the translation, adaptation and validation of the Diabetes Attitudes Scale - third version instrument into Brazilian Portuguese.

**Methods:**

methodological study carried out in six stages: initial translation, synthesis of the initial translation, back-translation, evaluation of the translated version by the Committee of Judges (27 Linguists and 29 health professionals), pre-test and validation. The pre-test and validation (test-retest) steps included 22 and 120 health professionals, respectively. The Content Validity Index, the analyses of internal consistency and reproducibility were performed using the R statistical program.

**Results:**

in the content validation, the instrument presented good acceptance among the Judges with a mean Content Validity Index of 0.94. The scale presented acceptable internal consistency (Cronbach’s alpha = 0.60), while the correlation of the total score at the test and retest moments was considered high (Polychoric Correlation Coefficient = 0.86). The Intra-class Correlation Coefficient, for the total score, presented a value of 0.65.

**Conclusion:**

the Brazilian version of the instrument (Escala de Atitudes dos Profissionais em relação ao Diabetes Mellitus) was considered valid and reliable for application by health professionals in Brazil.

## Introduction

Health professionals can significantly contribute so that the person living with diabetes can achieve the objectives related to glycemic control^1^. However, it has been observed that the practices of these professionals are still eminently prescriptive, being influenced, in the majority of cases, by the attitudes that they have in relation to the diabetes condition^2^ ^-^ ^3^.

Studies have identified the greatest trend of health professionals to be the adoption of a paternalistic attitude regarding decisions related to the treatment, with the justification that they know what is best for the person with the condition of diabetes^3^ ^-^ ^4^. In contrast, studies have shown the importance of the participation and empowerment of people living with this condition for achieving adequate outcomes and preventing complications related to diabetes^4^ ^-^ ^5^.

As the attitudes of the professionals determine the behavior they adopt^6^ ^-^ ^8^ and how they interact with people who have diabetes, causing repercussions in the treatment outcomes, it is necessary to identify the attitudes of these professionals when faced with this condition^9^. By identifying these attitudes, it is possible to establish educational strategies that contribute to a professional practice that considers the integrality of the care and the life context of the person with diabetes^7^ ^-^ ^9^. Therefore, valid and reliable instruments need to be used to measure the attitudes of these professionals, which also allow the results of research conducted in different countries to be compared.

Among the instruments available in the literature^9^ ^-^ ^10^, the Diabetes Attitudes Scale - third version (DAS-3) is the instrument which has the broadest spectrum of dimensions to assess the attitudes of health professionals in relation to diabetes mellitus. The construction of this instrument was guided by the Theory of Planned Action^9^. According to this theory, the intention of a person to perform certain behavior can be measured through the attitudes. The attitudes, in turn, are measured indirectly through the beliefs verbalized by the people, being able to strongly predict the behaviors that they adopt^7^. 

The DAS-3 consists of 33 questions divided in five related subscales: 1) need for special training to conduct educational interventions; 2) seriousness of Type 2 Diabetes; 3) value of strict glucose control for diabetes care; 4) psychosocial impact of diabetes on the lives of people and 5) autonomy of the person with diabetes^9^. It should be noted that the DAS-3 went through an evaluation process with 1,430 health professionals, proving to be valid and reliable, and has been translated and adapted to other countries, with the ability to maintain the original characteristics to measure the construct analyzed^9^ ^,^ ^11^ ^-^ ^12^.

In order to provide an instrument for use in the Brazilian context, this study aimed to carry out the translation, adaptation and validation of the Diabetes Attitudes Scale - third version (DAS-3).

## Method

This methodological study followed the recommendations established in the literature^13^. In the analysis of the conceptual equivalence and items, concepts related to diabetes and to the attitudes construct were explored in order to verify whether the dimensions of the instrument are relevant to the Brazilian cultural context. Considering the viability and relevance of using DAS-3 in Brazil, the following steps were performed. 

The translation was carried out independently by two translators, generating the T1 and T2 versions in Brazilian Portuguese. The translated versions were then compared by the same two translators and a third translator, which gave rise to a consensus version (T1-2). Next the instrument was back-translated to its original language, independently, by two other translators, in order to verify the concordance between the original version and the consensus version (T1-2)^13^.

After these steps, 30 health professionals and 30 from the field of Applied Linguistics were invited to participate as the Committee of Judges^13^. This was a convenience sample. The invitation was sent by e-mail and a link provided for access to the instrument previously uploaded to the web e-Surv platform. The judges were divided into three groups so that each group evaluated 11 statements, since the review of all 33 questions would take longer than 45 minutes. All the participants evaluated the instructions of the instrument and response options so that there was no impairment in the understanding and evaluation of the translated version. The aim was to evaluate the semantic, idiomatic, conceptual and experiential equivalences.

When comparing the original and the translated version, the judges evaluated the instrument according to the need for retranslation (1 = requires complete retranslation; 2 = requires partial retranslation with many changes; 3 = requires partial retranslation with a few changes; 4 = does not require retranslation) and the relevance of the reduction of the response options (from five options to four options). 

After obtaining the responses of the judges, the Content Validity Index (CVI) was calculated, defined by the sum of the relative frequencies of the “3” and “4” responses^14^. The assumption that the higher the CVI, the lower the number of changes needed to improve the text was considered.

A total of 22 health professionals that provided care to people with diabetes mellitus participated in the pre-test stage. In this stage, the questionnaire was sent electronically, and the link to access the instrument was provided. The professionals were asked to respond to the 33 statements of the instrument, to evaluate each statement for ease of understanding and clarity of the information and to present suggestions for improvement of the text^13^ ^-^ ^14^. 

Finally, in order to verify its validity and reliability, the instrument was applied, through the web e-Surv platform, with health professionals on two occasions with an interval of 15 days between the test and retest^14^. 

To calculate the sample size, a psychometric property was chosen that involves both the moment of the test and of the retest, the temporal reproducibility, and an alternative to its measure, the linear correlation. Thus, a significance level of 5%, test power of 80%, standard deviation equal in the test and retest scores and a correlation coefficient of 0.30 (minimum value to be detected in the evaluation of reliability) were considered. The minimum sample size required was 82 professionals. When considering a 20% losses, the final sample size required was 100 health professionals. 

The selection of the professionals was performed by convenience from the database of the project entitled “Measurement instruments for educational practices in chronic disease: interdisciplinarity and innovation”. Each professional that agreed to take part in the study was asked to indicate other professionals that worked with people who have diabetes. The application of the instrument was conducted in March and April 2016. 

The descriptive analysis of the categorical variables was performed by calculating the absolute and relative frequencies and, for the quantitative variables, the means, standard deviation, and percentiles were calculated. The evaluation of the internal consistency was made from the calculation of Cronbach’s alpha^15^ *.*

In the analysis of the reliability of the instrument, the Polychoric Correlation Coefficient was used, as the response scale is of the categorical ordinal type^16^. As with the Pearson’s linear correlation coefficient, the polychoric correlation coefficient can have values ​​between -1 and 1. The stronger correlations relate to coefficient values closer to -1 (negative correlations) or 1 (positive correlations). Polychoric correlation coefficient values near zero indicate weak or no linear correlations. The percentage of concordance between the responses in the test-retest was calculated to support the decision regarding the temporal stability of the instrument.

The Intra-class Correlation Coefficient (ICC) was also used as a measure of concordance between the total score obtained in the two applications of the instrument, while the Wilcoxon test was used to verify whether there was a statistical difference between the median score of the first and second application of the instrument^11^. Data analysis was carried out using the R^†^ statistical program. The significance level considered for the statistical tests was 5%. 

The study was approved by the Research Ethics Committee of the Federal University of Minas Gerais (Authorization No. 1.072.984). The consent form was made available electronically on the first page of the questionnaire, where the professionals recorded their agreement to participate in the study.

## Results

From the 60 invitations sent to the sample of professionals selected to participate in the Committee of Judges, 56 completed questionnaires were obtained, 29 completed by the health professionals (51.8%) and 27 by the linguists (48.2% ). A total of 3.7% of the judges reported *Lato sensu* post-graduate level education and 80.3% reported having performed a *Stricto sensu* post-graduate course. 

In general, the instrument presented high levels of CVI, resulting in a mean CVI of 0.94, with a standard deviation of 0.09. Statements 16 and 27, however, presented the lowest CVI values, indicating the need for further changes, as shown in Table 1. 

**Table 1:** Absolute and relative frequencies of the responses of the Committee of Judges in the evaluation of the instrument items and content validity index. Belo Horizonte, MG, Brazil, 2015

Item	Requires complete retranslation	Requires partial retranslation with many changes	Requires partial retranslation with a few changes	Does not require retranslation	CVI^*^
	N (%)^†^				
Instructions	0	5 (8.9)	20 (35.7)	31 (55.4)	0.91
Response options	1 (1.8)	1 (1.8)	19 (33.9)	35 (62.5)	0.96
1	0	2 (11.1)	9 (50.0)	7 (38.9)	0.89
2	0	0	8 (44.4)	10 (55.6)	1,00
3	0	0	8 (44.4)	10 (55.6)	1,00
4	0	0	3 (16.7)	15 (88.3)	1.00
5	0	0	6 (33.3)	12 (66.7)	1.00
6	0	0	6 (33.3)	12 (66.7)	1.00
7	0	0	1 (5.6)	17 (94.4)	1.00
8	0	0	1 (5.6)	17 (94.4)	1.00
9	0	1 (5.6)	4 (22.2)	13 (72.2)	0.94
10	0	0	9 (50.0)	9 (50.0)	1.00
11	0	0	11 (61.1)	7 (38.9)	1.00
12	0	0	8 (40.0)	12 (60.0)	1.00
13	0	3 (15.0)	5 (25.0)	12 (60.0)	0.85
14	1 (5.0)	1 (5.0)	11 (55.0)	7 (35.0)	0.90
15	0	2 (10.0)	9 (45.0)	9 (45.0)	0.90
16	7 (35.0)	2 (10.0)	4 (20.0)	7 (35.0)	0.55
17	0	0	7 (35.0)	13 (65.0)	1.00
18	1 (5.0)	0	5 (25.0)	14 (70.0)	0.95
19	0	0	7 (35.0)	13 (65.0)	1.00
20	0	1 (5.0)	3 (15.0)	16 (80.0)	0.95
21	0	0	0	20 (100.0)	1.00
22	0	1 (5.0)	4 (20.0)	15 (75.0)	0.95
23	1 (5.0)	3 (15.0)	4 (20.0)	12 (60.0)	0.80
24	0	3 (16.7)	11 (61.1)	4 (22.2)	0.83
25	0	0	3 (16.7)	15 (83.3)	1.00
26	0	0	4 (22.2)	14 (77.8)	1.00
27	1 (5.6)	3 (16.7)	10 (55.6)	4 (22.2)	0.78
28	0	0	7 (38.9)	11 (61.1)	1.00
29	0	2 (11.1)	7 (38.9)	9 (50.0)	0.89
30	0	0	12 (66.7)	6 (33.3)	1.00
31	0	1 (5.6)	9 (50.0)	8 (44.4)	0.94
32	0	0	6 (33.3)	12 (66.7)	1.00
33	0	0	1 (5.6)	17 (94.4)	1.00
Mean CVI (SD)			0.94 (0.09)		

*CVI - content validity index; †The relative frequencies sum to 100% within the lines and absolute frequencies correspond to the number of evaluator Judges for each group of statements of the instrument, with 18 of them assessing questions 1 to 11; 20 judges assessing questions 12 to 23; and 18 judges assessing questions 24 to 33. All the judges reviewed the instructions and instrument response options.

The reduction of response options to four alternatives was evaluated as relevant by the judges and by the health professionals. The reasons given were: ease of choice and understanding of the answer choices among people who would respond to the instrument; no significant difference within the Brazilian cultural context between the options, “disagree” and “totally disagree”. 

In order to preserve the comparison between the scores obtained with the original instrument and the instrument translated and adapted in Brazil, it was decided to maintain the score of response options with the range between 1 and 5 points. Thus, the following points were awarded to the statements with scores in direct order: disagree - 1 point, no opinion - 3 points, partially agree - 4 points, agree - 5 points. Regarding the statements that have reversed scores (2, 3, 7, 11, 13, 15, 16, 23, 26 and 28), the points were distributed as follows: agree - 1 point, partially agree - 2 points, no opinion - 3 points and disagree - 5 points. It is important to note that the “no opinion” option is scored the same in direct and reverse order.

**Figure 1:**
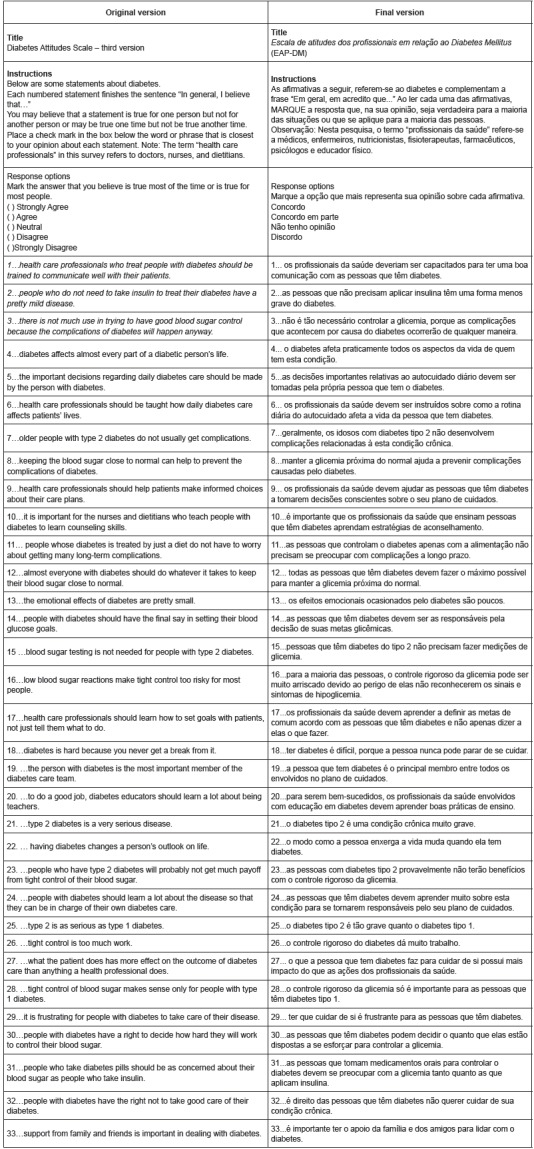
Description of items from the original version of the Diabetes Attitudes Scale - third version and the Brazilian version of the *Escala de Atitudes dos Profissionais em relação ao Diabetes Mellitus*, Belo Horizonte, MG, Brazil, 2015

The main changes made in the translated version after the suggestions given by the judges and in the pretest phase were: (1) replacing the term “patient”, “user” and “diabetic” with “person with diabetes”; (2) inclusion of physiotherapy, pharmacy, physical education and psychology professionals; (3) changing the expression “self-care plan” to “care plan” and (4) replacing the word “disease” with “chronic condition”. After these steps, the final version of the *Escala de Atitudes dos Profissionais em relação ao Diabetes Mellitus* (EAP-DM) was obtained, as presented in Figure 1.

A total of 120 health professionals participated in the validation step (test-retest). The characterization of the participants is presented in Table 2.

**Table 2:** Characterization of the professionals that participated in the validation stage of the EAP-DM. Belo Horizonte, MG, Brazil, 2016 (n=120)

Profile of the participants		n (%)*
Gender		
	Female	103 (85.8)
	Male	17 (14.2)
Area of qualification		
	Nursing	64 (53.3)
	Medicine	35 (29.2)
	Nutrition	12 (10.0)
	Physiotherapy	4 (3.3)
	Physical Education	3 (2.5)
	Pharmacy	1 (0.83)
	Psychology	1 (0.83)
Level of practice		
	Primary	40 (33.3)
	Secondary	18 (15.0)
	Tertiary	15 (12.5)
	Primary and Secondary	14 (11.7)
	Primary and Tertiary	9 (7.5)
Level of practice		
	Secondary and Tertiary	15 (12.5)
	Primary, Secondary and Tertiary	9 (7.5)
Qualification		
	Master’s degree	41 (34.2)
	Doctoral degree	33 (27.5)
	Specialization	32 (26.7)
	Bachelors degree	14 (11.7)
Sector of practice		
	Public	69 (57.5)
	Private	10 (8.3)
	Public and private	41 (34.2)
Region of the country		
	Southeast	83 (69.2)
	Central-east	15 (12.5)
	South	12 (10.0)
	Northeast	10 (8.3)
Years of experience - Median (min-max)		8.0 (1.0-45.0)

* n (%): Absolute and relative frequencies

The overall Cronbach’s alpha value for the *Escala de Atitudes dos Profissionais em relação ao Diabetes Mellitus* was 0.60, indicating acceptable internal consistency. 

Table 3 shows the presence of moderate to high correlations between the items at the test and retest moments.

**Table 3:** Correlation between the responses to the items, between the scores in the subscale and total score in the test and retest and Cronbach’s alpha Coefficient (α) for the *Escala de Avaliação das Atitudes dos Profissionais em relação ao Diabete* *s* (EAP-DM). Belo Horizonte, MG, Brazil, 2016 (n=120)

Subscale and items	Polychoric Correlation Coefficient - test and retest	Cronbach’s alpha for the subscales and overall scale	Percentage of concordance between the responses in the test and retest
Needs for professional training	0.987	0.57	
Question 1	0.813		97.5
Question 6	- 0.894		97.5
Question 10	0.768		95.0
Question 17	0.731		87.5
Question 20	0.778		94.2
Seriousness of Type 2 Diabetes Mellitus	0.919	0.54	
Question 2	0.811		72.5
Question 7	0.708		91.2
Question 11	0.593		91.6
Question 15	0.517		89.2
Question 21	0.682		67.5
Question 25	0.686		74.2
Question 31	0.678		83.3
Importance of strict glucose control	0.900	0.55	
Question 3^†^	---		99.2
Question 8	0.623		88.3
Question 12	0.763		78.3
Question 16	0.679		69.2
Question 23	0.674		94.2
Question 26	0.800		74.2
Question 28	0.631		91.6
Psychosocial impact of diabetes	0.912	0.58	
Question 4	0.794		82.0
Question 13	0.466		92.5
Question 18	0.692		70.0
Question 22	0.618		56.6
Question 29	0.521		65.8
Question 33^†^	---		99.2
Importance of autonomy	0.891	0.58	
Question 5	0.642		69.2
Question 9	0.587		95.8
Question 14	0.659		61.6
Question 19	0.565		75.8
Question 24	0.574		82.5
Question 27	0.443		70.0
Question 30	0.653		66.6
Question 32	0.752		65.8
Overall score	0.860	0.60*	

*Overall alpha; ^†^ The responses to the question do not show variability in at least one of the moments, with the calculation of the correlation coefficient not being possible

The reliability analysis of the instrument was supported by calculating the Intra-class Correlation Coefficient, which indicated moderate concordance in all subscales and in the general scale, as presented in Table 4.

**Table 4:** Intra-class correlation coefficient for the overall scale and its subscales. Belo Horizonte, MG, Brazil, 2016 (n=120)

Overall scale and subscales	Intra-class correlation coefficient (95%)
Needs for professional training	0.54 (0.40-0.66)
Seriousness of Type 2 Diabetes Mellitus	0.67 (0.56-0.76)
Importance of strict glucose control	0.58 (0.45-0.69)
Psychosocial impact of diabetes	0.68 (0.57-0.76)
Importance of autonomy	0.67 (0.56-0.76)
General scale	0.65 (0.54-0.75)

## Discussion

Opting to culturally adapt an instrument is due to the various advantages already mentioned by the literature, such as savings time and the possibility of comparing the results with studies carried out in other countries^13^. 

The studies that translated and adapted the DAS-3 used methodology similar to that presented in this study, differing only in the composition of the specialists that composed the Committee of Judges. Despite methodological differences related to the performance of the Committee of Judges, the DAS-3 has proved to be a valid, reliable, and easy to understand instrument, for use by professionals in different countries^9^ ^,^ ^11^ ^-^ ^12^.

The main changes in the items of the translated version were related to the change of terms used to describe people who have diabetes and the reduction of the response options. The term “diabetic” is no longer used, due to the current principles that consider the importance of the autonomy of people living with the condition of diabetes in the process of choices in their care plan. The term “diabetic”, used as a noun, labels people who have diabetes from a negative perspective and also implies that all people living with this condition are equal, resulting in the establishment of standardized behaviors that do not consider the life story and the individual needs of these people^17^. 

The reduction of the response options should also be highlighted, which was considered relevant by the majority of the specialists. The justifications of the judges for the reduction of response options were related to the discussions presented in the international literature, which demonstrate the existence of differences in response patterns for Likert type scales among people with different education and cultures^18^.

The results of the evaluation of the psychometric properties indicated adequate internal consistency. Other studies found the presence of variation in the alpha values, which is justified by the instrument being applied in populations with different characteristics. Nevertheless, the versions translated and validated in other countries have also obtained internal consistency considered adequate^9^ ^,^ ^11^ ^-^ ^12^.

The median score of the retest can be considered equal to the median score of the test for the majority of the subscales. It should be noted that the differences in medians found for the overall score and the “psychosocial impact of diabetes” subscale, although significant, can be considered small (0.04 and 0.14 points respectively). The scores for each subscale were found to be similar to the results of a study conducted in Spain^11^.

A moderate to high discrimination capability was observed for the items, verified by the Polychoric Correlation Coefficients ranging from 0.443 to 0.813. It was not possible to compare these coefficients with studies performed in other countries, since these studies did not use the Polychoric Correlation Coefficient. 

In the analysis of the reliability through the stability, an ICC of 0.65 was obtained for the entire scale, demonstrating the temporal stability of the instrument^11^.

It is worth considering that evidence of validity should be accumulated to strengthen confidence in the use of scales. Therefore, it is suggested that this scale be applied with representative and more heterogeneous samples of health professionals, considering the different occupational categories and regions of the country.

## Conclusion

It was concluded that the Brazilian version of Diabetes Attitudes Scale - third version, with the name *Escala de Atitudes dos Profissionais em Relação ao Diabetes Mellitus* (EAP-DM), fulfilled the criteria of equivalence between the original instrument and the translated version, demonstrating its validity and reliability for evaluating the attitudes of health professionals in relation to diabetes. The application of this instrument may help in the comprehension of care practices directed toward people who have diabetes and thus subsidize training programs that target health professionals. 
